# Exposure to constant artificial light alters honey bee sleep rhythms and disrupts sleep

**DOI:** 10.1038/s41598-024-73378-9

**Published:** 2024-11-12

**Authors:** Ashley Y. Kim, Aura Velazquez, Belen Saavedra, Benjamin Smarr, James C. Nieh

**Affiliations:** 1 School of Biological Sciences, Section of Ecology, Behavior, and Evolution, University of California San Diego, 9500 Gilman Drive, La Jolla, CA 92093 USA; 2 Facultad de Ciencias Químicas, Universidad La Salle México, Benjamín Franklin 45, Ciudad de México, 06140 México; 3 Computer and Information Science, Berea College, 101 Chestnut Street, Berea, KY 40403 USA; 4 Shu Chien - Gene Lay Department of Bioengineering & Halicioğlu Data Science Institute, University of California San Diego, 9500 Gilman Drive, La Jolla, CA 92093 USA

**Keywords:** Light pollution, Anthropogenic stressors, Circadian rhythm, Sleep disturbances, Honey bee colony health, Ecology, Animal behaviour, Entomology

## Abstract

Artificial light at night (ALAN) changes animal behavior in multiple invertebrates and vertebrates and can result in decreased fitness. However, ALAN effects have not been studied in European honey bees (*Apis mellifera*), an important pollinator in which foragers show strong circadian rhythmicity. Colonies can be exposed to ALAN in swarm clusters, when bees cluster outside the nest on hot days and evenings, and, in limited cases, when they build nests in the open. We captured and maintained foragers in incubated cages and subjected them to constant light (LL), constant dark (DD), or 12 h light:12 h dark (LD) cycle, and observed them with infrared cameras. After 79 h, there was a significant interaction of treatment and time because LL bees slept less. In detail, the bees maintained a regular sleep pattern for three days but LL bees showed a shift on the fourth day. LL bees had the largest sleep differences from LD controls, with trends of lengthened periods and increased phase misalignment from both LD and DD bees. LL bees also experienced significantly more disturbances from their nestmates and preferred to sleep in the lower portion of the cages, which had significantly lower light intensity. These findings suggest that ALAN can disrupt the sleep of honey bee foragers, which has implications for their behavior and overall colony health.

## Introduction

Insect declines are historically thought to be driven by climate change, habitat loss, introduced species, and agricultural pesticides^1^. Although a neglected stressor, light pollution covers roughly a quarter of the Earth’s surface^2^ and disturbs biological rhythms, orientation, and animal mating behavior and reproductive success^3^. As biodiversity continues to decline due to anthropogenic disturbances, understanding the effects of light pollution on insect systems is increasingly crucial for conservation. Artificial Light at Night (ALAN) has ecosystem impacts^4^ because it can interfere with pollination networks and disrupt plant fitness and food webs^5^. For example, ALAN can significantly decrease the amount of time that moths spend feeding, which could contribute to their decline and negatively impact the pollination services they provide^6^. However, the underlying effects of ALAN on pollinator behavior are relatively unknown^4^, particularly for insects such as social bees in which cooperative foraging is essential for colony fitness.

In general, honey bees prefer to nest in dark cavities where the only light source would be the hive entrance. Nurse honey bees live inside a normally dark hive and do not show strong circadian rhythms until they become foragers, when they are regularly exposed to daylight, an important zeitgeber^7^. However, if ALAN increases, the nest entrance light source can be constant during a 24-hour period or create illumination that disrupts the natural day/night cycle. Another source of potential disruption involves bee light sensitivity. When honey bees travel to a dark part of the nest, the light sensitivity of their eyes can increase by up to 1000-fold^8^. This photic adaptation may be disrupted when constant light illuminates parts of the hiveor if there are insufficient dark spaces within the nest.

There are multiple ways in which honey bees can be exposed to artificial light if they are outside their enclosed nests and in an environment with artificial light. Honey bees can cluster outside the nest during extreme heat, a phenomenon known as “bee bearding”, typically during hot (≥ 38 °C) and humid nights and days that occur from mid-June to August in areas such as Europe^9^. This response to extreme heat stress is part of colony thermoregulation^10^ and may occur more frequently as global temperatures increase^11^. Honey bee colonies (*Apis* species) also reproduce through swarming, a process in which honey bee colonies split into two or more colonies as a means of reproduction. The swarming process typically takes hours to complete but can also span multiple days^12^. The frequency of swarms in urban environments with ALAN rises when fall and winter precipitation increases in southwestern deserts^13^.

**Fig. 1:**
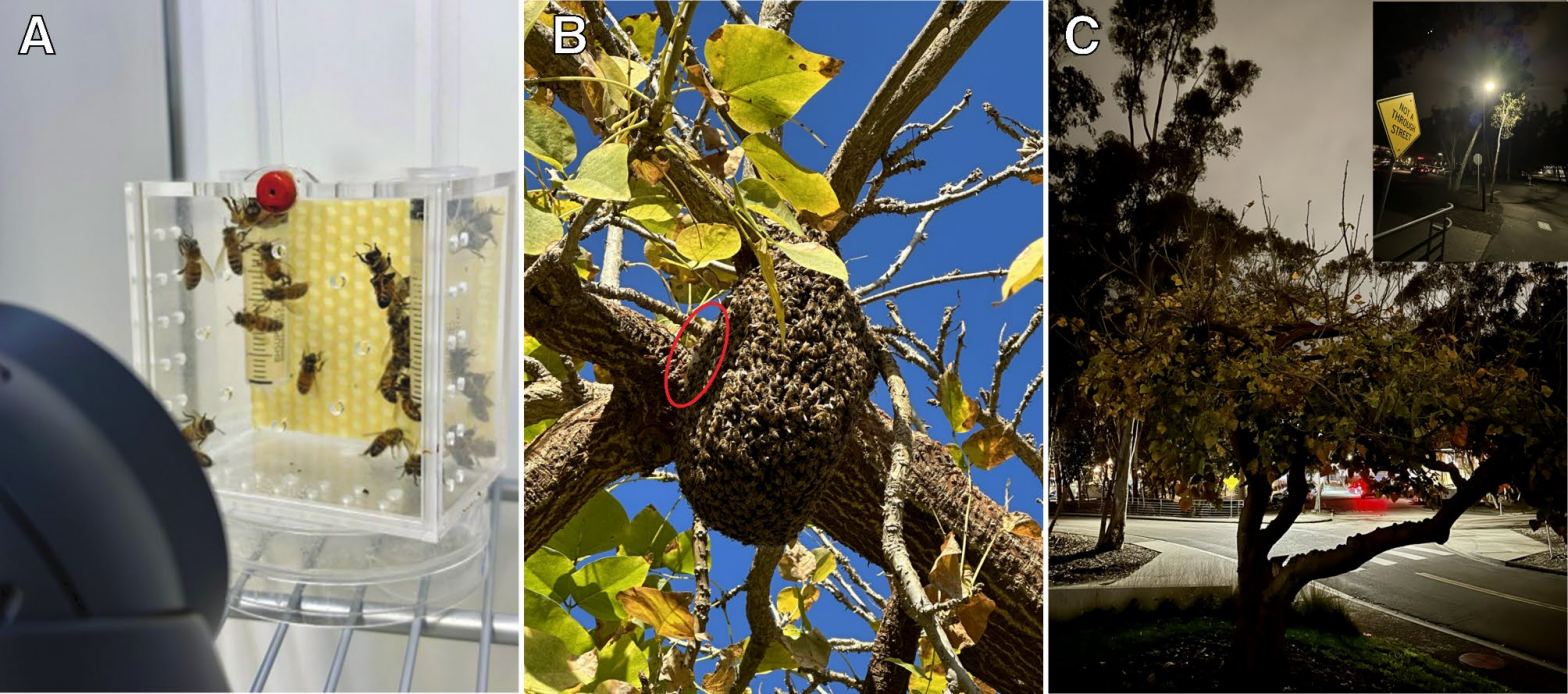
The design of our experiment and an example of bees exposed to ALAN. (A) A typical cage with a beeswax foundation, feeding syringes, and queen mandibular pheromone as used in our experiment. (B) A feral *A. mellifera* colony nesting outside on the UC San Diego campus. This colony is not a swarm because it has built several wax combs (red oval surrounds exposed yellow comb). (C) This colony is exposed to ALAN as shown in this nighttime photo of the tree in which the colony is nesting and the inset, which shows the streetlight behind the tree (note the position of the yellow sign in both photos). Although such open nesting is rare, it can occur (see Introduction).

Finally, bees (*Apis mellifera*, *Apis cerana*, *Apis nigrocincta*, and *Apis koschevnikovi*) can be exposed to ALAN when they build nests in the open, not inside cavities (Fig. 1). Although *A. mellifera* is a cavity-nesting species, beekeepers and others have reported cases in temperate climates in which colonies have not nested inside a cavity but, instead, in the open where they can be exposed to ALAN. Winston^14^ observed that, in the tropics, feral *Apis mellifera* colonies, including *Apis mellifera scutellata* can nest in the open, particularly in drier habitats. These colonies can be found suspended from tree limbs, groups of small branches, or rocks. Winston et al.^15^ published, as a personal observation, that 20% of the feral honey bee nests (likely *scutellata*-hybrids) in Venezuela were built in the open. Ratnieks et al.^16^ observed that out of 30 feral nests (also likely *scutellata*-hybrids) in Tapachula, Mexico, three were built in the open (10%). Boreham and Roubik^17^ reported that 11% of *scutellata*-hybrid nests were made in the open on vegetation.

Honey bee circadian rhythms are free running under both constant light or dark conditions, can be entrained by light-dark (LD) cycles, can be phase-shifted by pulses of light, and can vary from 20 to 26 h cycles^18^. These rhythms also help regulate sleep, which can be separated by stages of light and deep sleep, characterized by extended total durations (or bouts) of antennal immobility^19^. Sleep in honey bees is a dynamic process that consists of relative immobility, the metasoma pumping discontinuously (discontinuous ventilation), and an increased response threshold to external stimuli. A sleeping bee is generally immobile, but its legs, tarsi, or antennae can twitch, and its body can exhibit subtle movements caused by nestmates disturbing the sleeping bee. Bees need sleep to effectively communicate with nestmates via the waggle dance^20^. Sleep is therefore important for colony fitness because honey bee dance communication can significantly enhance colony fitness^21^.

We hypothesized that foragers collected from colonies will maintain, for some time, their bee sleep behavior, under the normally dark conditions inside the nest, but would have these sleep rhythms disrupted when they are exposed to constant illumination. We also hypothesized that bees under constant light illumination would experience greater disruption from nestmates and prefer to sleep under lower light levels, when possible.

## Methods

### General setup

In Spring 2021 and 2024, we conducted eight trials (*n* = 16 colonies) total to observe sleep behavior under both dark /light treatments (5 trials) and 12 L:12D (3 trials). In total, we tested 281 bees across all trials, and each cage contained 14–28 individuals. To account for the variance of the number of individuals per cage in 2021 trials, we used 23 individuals per cage for all 2024 trials. In La Jolla, California, we captured *Apis mellifera ligustica* foragers from sucrose solution feeders (2.5 M) at hive entrances and transported the bees from the UCSD Biology Field Station (BFS) to the lab. Foragers were thus exposed to natural cycles of light and dark for multiple days before their capture. Foragers were placed into two different experimental cages. The experimental cages were made from transparent acrylic (12 cm long × 8 cm wide × 12 cm high) and had a sliding door on the top with holes for ventilation with another two holes to insert two 5 mL syringes containing 2.5 M sucrose solution (Fig. 1A). These syringes were placed close to the beeswax comb foundation but did not touch the foundation. Bees could feed from these syringes *ad libitum* and, based on our video recordings, had no difficulty in accessing this sucrose solution. We also placed a half strip of a queen mandibular pheromone (QMP, Temp Queen Pheromone, catalog number DC-705, Meyer Bees, Chicago, USA) because queen presence, which can be simulated by QMP, is required for normal worker activity^22^. The honeycomb wax foundation was placed on the back wall behind the sucrose solution feeders to provide a place for bees to congregate, and, based on our videos, we observed that the majority of bees spent most of their time on this comb foundation. We used wax foundation instead of bee-built comb to provide a standard, uniform surface for bees to congregate on. The experimental cages were placed inside an incubator (24 °C, 50% relative humidity) and bee behaviors were recorded with two infrared-sensitive Lorex 4K cameras (LNE894AB) connected to a digital recording system (Lorex N842, Lorex USA) for 24 h for five consecutive days. Each cage was video recorded by one camera, which had a full view of all bees in the entire cage. Moderate-intensity infrared LED lights (iThird Remote Control LED Light Bulbs, 850 nm), which are invisible to bees because they cannot typically detect light wavelengths higher than 550 nm^23^, were used to illuminate the bees in both cages for video recordings.

To simulate constant illumination from buildings and streetlights, white LED light bulbs were used with a brightness of 2.7 ± 0.1 µmol/m^2^/s (measured inside at the level of the cage bottom^24^. We placed the light treatment cage in a light-proof box made of cardboard and aluminum foil and had an exposed LED bulb (light treatment). The dark treatment cage was placed into the incubator and had no exposure to constant light. To determine levels at the top and bottom of the cage, we made 15 measurements of photon flux at the top and 15 measurements of photon flux at the bottom of the cage. We measured photon flux with an ePAR light sensor (Apogee Instruments, Model DLI-600, spectral range of 390–760 nm) placed inside the cage and flush with the cage top and, separately, on the cage floor by cutting a circular hole into an identical cage in which we inserted the top of the light sensor, level with the cage floor and pointed up at the cage top through which light entered.

We exposed bees to a third condition as a control, 12 h of light and 12 h of dark each 24 h (3 total trials), because we wished to compare the effects of foragers continuously exposed to light for five days, as could occur when colonies form bee beards^9^ or swarm or nest in the open near artificial light. A 12 h light:12 h dark cycle is a typical light cycle that bees can experience and is a standard cycle for circadian experiments. In addition, honey bees may remain in dark conditions for prolonged periods, either as a result of overwintering inside the colony^25^ or in response to poor weather^26^. We, therefore, simulated a situation in which bees were kept in the constant dark for five days (dark situation).

To score the sleep data, we looked at the first five minutes of each hour to count how many foragers were sleeping that displayed all three sleep markers (antennal immobility, non-continuously pulsating abdomen, and leg immobility) and the number of foragers alive or dead on each recorded day. We chose a five-minute time interval that included our sleep markers based on standard methods for observing honey bee sleep^27^ and fruit fly sleep^28^. Observers were trained to count forager bees sleeping on the comb and the sucrose syringe. We defined bee sleep as antennal immobility and remaining completely stationary for the entire five-minute observation period.

### Potential sleep disruptors

Based upon visual inspection of the data, we detected a clear divergence in the number of disruptions received by sleeping bees after 90 h in the constant light condition as compared to the constant dark condition. We, therefore, analyzed the video recordings after this time point for evidence of light avoidance or sleep disruption. Observers who had carefully watched and scored a total of 107.3 h of video (first five minutes of each hour within a 24 h period) for bee sleep noted two potential sleep disruptors differed between the dark and light treatments: (1) bees sleeping on the bottom of the cage, where light levels were significantly lower (see Results) or (2) disturbance of a sleeping bee by a moving nestmate. Within the first 5 min of each hour, observers therefore (1) scored if the bee was sleeping in the upper or lower 50% of the cage, and (2) counted the number of times each sleeping bee (see definition above) was moved by physical contact with a non-sleeping bee passing by. We defined a disturbance as a sleeping bee moving after it was contacted by another bee. If a bee was disrupted by another bee and woke up, we would still count it as sleeping within the five-minute interval if the bee went back to sleep within less than 30 s.

### Statistics

To examine the effects of treatment, cumulative time since the start of the trial (both fixed effects), and their interaction (with cage identity a random effect nested within treatment) on the log-transformed proportion of sleeping bees, we used Repeated Measures Mixed Models (REML algorithm). To compare *the light levels* in the upper and lower portion of the cage, we used a *t*-test comparing light levels in both cage sections. To analyze the effects of potential behavioral sleep disruptors we used Repeated-Measures Mixed Models (REML algorithm) with cage ID nested within treatment (light or dark) as a random effect, cumulative time from the start of the experiment as the time effect (fixed effect), and the interaction of cumulative time x treatment (fixed effect). For these models, we used JMP Statistical Software v16.0.0.

For all other analyses, we used Matlab 2024a. To analyze sleep rhythmicity, the proportion asleep was aligned across nests by hours from the experiment onset. Replicates were grouped by conditions (constant light, constant dark, and light: dark). In the constant light and constant dark conditions, two older replicates were replaced with two new replicates for two reasons. First, we observed a cohort effect on survival that was larger than the effect of the condition, necessitating the generation of new data under more uniform conditions to eliminate this effect. Second, there was high mortality in two nests in both constant light and constant dark, resulting in no activity or only a single individual still alive during the last two days. This made the analysis of period shift and period assessment infeasible (*n* = 2 cycles only per nest). The new cohorts completed all days of the experiment, allowing for a total of *n* = 3 nests per condition.

Differences in circadian rhythmicity were analyzed using several methods. The period was assessed by performing a Fourier transform on the percentage of time asleep and calculating tau (τ) from the beta coefficient. Alignment was determined by subtracting the percentage asleep values of the light: dark control group from each experimental condition in pairs by nest (e.g., light: dark 1-constant dark 1, light: dark 2-constant light 2, etc.).

We hypothesized that if the periods remained consistent across days, the area resulting from the subtraction would be smaller, indicating greater similarity between the two different curves. Conversely, if the period drifted over time, the two lines should align less well, even if both remained rhythmic, generating a larger difference value. Differences were calculated across all time points, and statistics and figures were generated from the results on the fourth day from the beginning of the experiment. This approach allowed maximal differences to accrue, reflecting alignment differences more accurately without being diluted by the initial days when changes had not yet accumulated.

The absolute values of the differences were also calculated to ensure that negative and positive results from the subtraction of curves did not cancel out, thereby avoiding artifactually lowered differences. Finally, differences in peak timing on the fourth day were calculated as a proxy for period differences by finding the distance in hours from the light: dark midpoint peak to the midpoint peak in the other two conditions. Comparisons were made using Wilcoxon rank-sum tests between differences from light: dark in constant light and differences from light: dark in constant dark conditions. These analyses were based on Smarr et al.^29^.

Survival analysis was conducted by comparing the percentage of bees still alive at the end of the experiment (120 h from onset). A Kruskal-Wallis test was employed to assess the effect of condition on the percentage of bees alive, with three replicates per condition.

## Results

### Proportion of bees sleeping

**Fig. 2:**
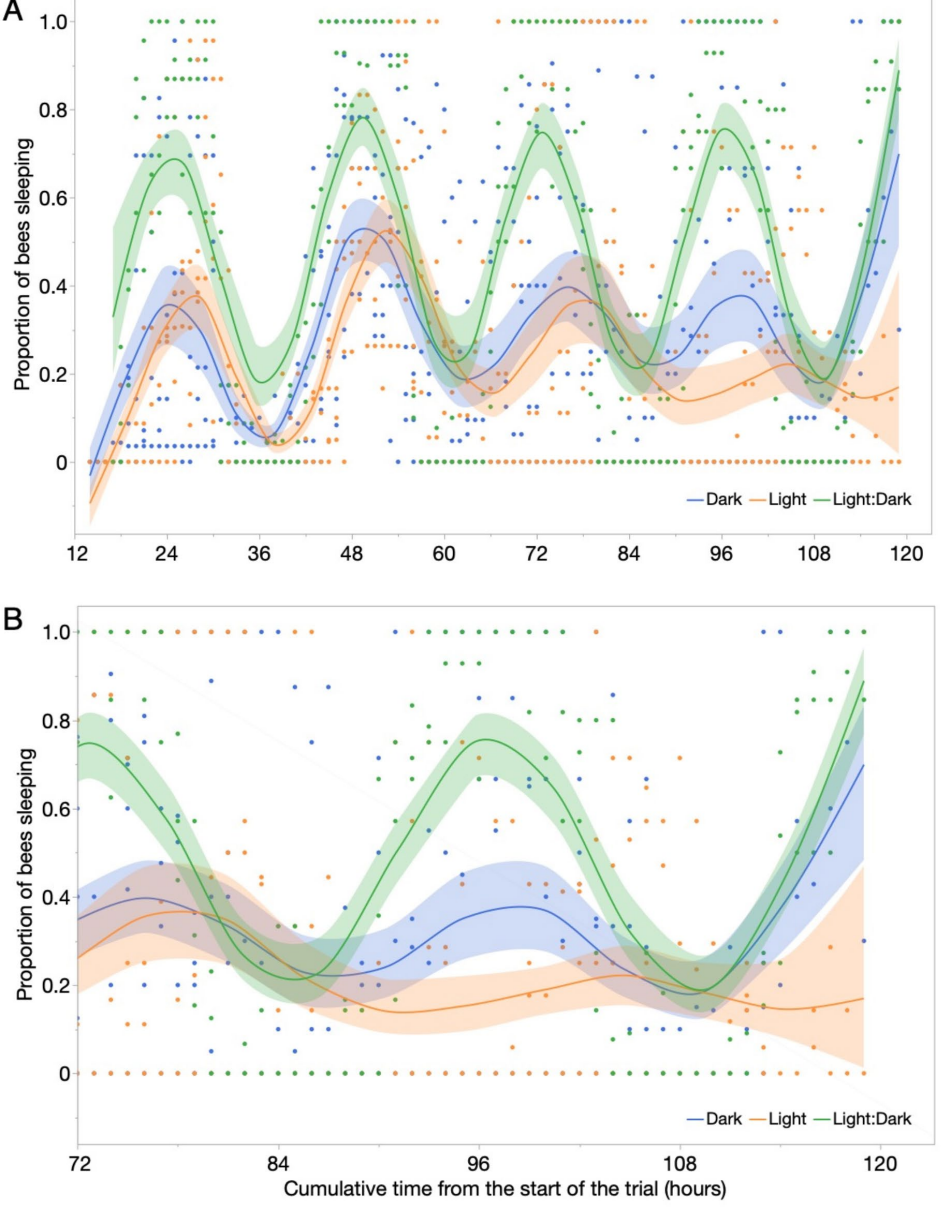
Bee sleep follows a roughly circadian pattern over the entire experiment, but changes after about 79 h in bees under constant illumination. The Light: Dark treatment refers to 12 h of light followed by 12 h of dark, a standard cycling. (A) Over the entire experiment, bee sleep changed significantly over time (*P* = 0.0003). (B) From 79 h until the end of the experiment, there was a significant interaction of treatment x time (*P* = 0.0017) because those exposed to constant light slept less than bees kept in the dark, the normal state inside the nest. During this interval, there was no significant effect of treatment on sleep (*P* = 0.76) and no significant effect of time (*P* = 0.73). All data points are shown, along with best-fit spline curves and 95% confidence intervals.

There was no significant effect of treatment (*F*_*2*, 10_ =1.99, *P* = 0.19), but a significant effect of cumulative time (*F*_1, 773_ = 13.19, *P* = 0.0003) on the proportion of bees sleeping. The interaction of treatment x cumulative time (*F*_*2*,770_ =2.07, *P* = 0.13) was not significant. In this model, cage accounted for only 2% of model variance. Around day 4 (79 h after the start of the trial), however, there was a crossover point between the two treatments. There was a significant interaction between treatment and time (*F*_*2*,246_ = 6., *P* = 0.0017) because bees exposed to constant light slept significantly less than bees in the dark treatment (Fig. 2B). There was no significant effect of treatment (*F*_2,8_ =0.28, *P* = 0.76) or time (*F*_1,246_ =0.12, *P* = 0.73) alone.

This analysis focused on the differences between the treatments, but not on the inherent circadian periodicity.

### Sleep rhythmicity

**Fig. 3:**
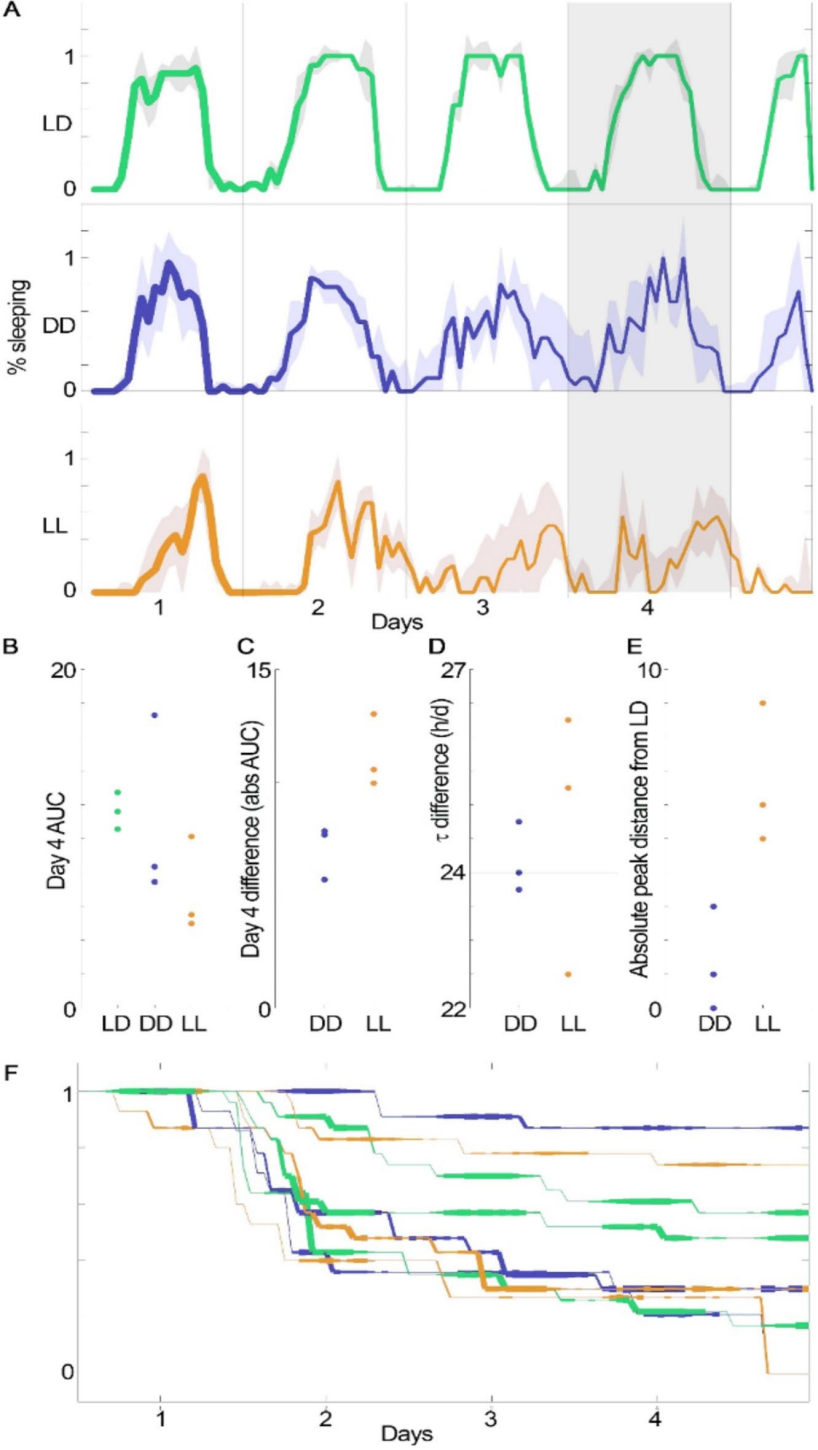
Sleep rhythms exhibited varying trends under different lighting conditions: constant light (LL, orange), constant dark (DD, blue), and 12-hour light:12-hour dark (LD, green) cycles. A) Compared to the control (LD), both DD and LL conditions showed a significant decrease in the percentage of time asleep by the fourth day (grey shaded area). The line represents the median across each condition, with the width proportional to the percentage surviving, and the fill indicating +/- 1 MAD (median absolute deviation). B-F) The area under the curve on the fourth day indicates high variance, with a trend of decreasing sleep in LL bees. Trends showing increased differences in LL from LD compared to DD from LD are illustrated in the absolute value of the percentage asleep AUC (area under the curve) difference (B), the period (τ) calculated from the Fourier transform (D), and the absolute value of peak distance in time from the LD peak (E). F) No significant differences in survival over time were noted. The line width is proportional to the percentage of surviving bees.

In analyzing the sleep rhythmicity over the entire trial duration, we observed a significant decrease in the percentage of bees asleep over time in both constant light and constant dark conditions as compared to 12 h light: 12 h dark condition (Kruskal-Wallis, *P* = 7 × 10^−8^; Fig. 3A). In this overall data, we also observed several convergent trends. Light treatment bees tended to sleep less (*P* = 0.1; Fig. 3C). Period analysis indicated a lengthening trend in the constant light condition (*P* = 0.1; Fig. 3D). Additionally, bees exposed to constant light showed a trend of increased peak distance with respect to light: dark treatment bees as compared to constant dark bees with respect to light: dark treatment bees (Fig. 3E). These trends collectively support a greater impact of constant light compared to constant dark. There was no significant difference in survival (*P* = 0.4; Fig. 3F).

### Potential sleep disruptors

**Fig. 4:**
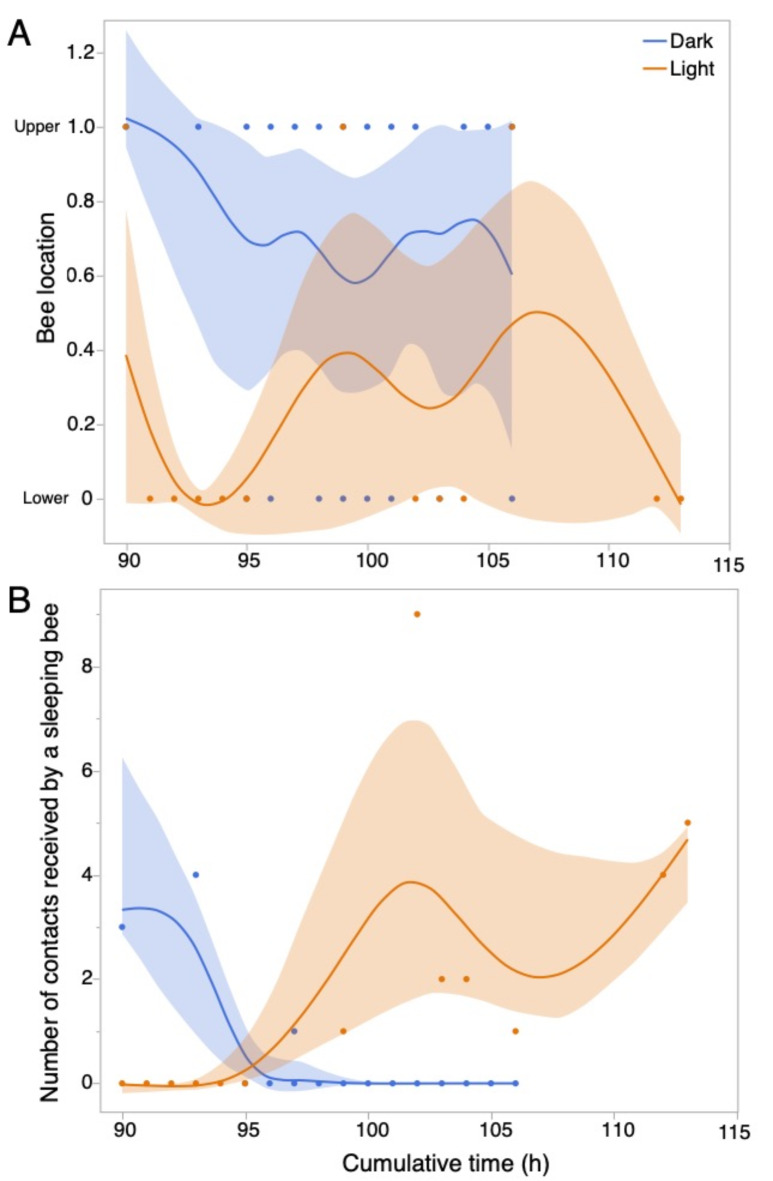
Potential markers of sleep disruptors related to light exposure. (A) Bees experiencing constant light slept significantly more often on the bottom of the cage as compared to bees experiencing constant dark at the end of the experiment (≥ 90 h). There was a significant effect of treatment (*P* < 0.001), but no significant effect of time or the interaction of treatment x time (*P* ≥ 0.49). (B) Bees in the light treatment received more potentially disruptive contacts than bees in the dark treatment. There were significant effects of treatment (*P* = 0.004) and the interaction of treatment x time (*P* < 0.001), which is shown in the different slopes of the spline curves over time (*P* < 0.001). All data points are shown, along with best-fit spline curves and 95% confidence intervals.

There was 7-fold more light in the upper as compared to the lower part of the cage (upper part of the cage: 18.2 ± 0.1 µmol/m^2^/s; lower part of the cage 2.7 ± 0.1 µmol/m^2^/s; t-test, *t*_28_ = 315.27, *P* < 0.0001). In the light treatment, bees slept significantly more often in the lower part of the cage where there was less light. Bees in the dark treatment slept higher up in their cages as compared to bees in the light treatment (treatment effect: *F*_1,34_ =19.62, *P* < 0.001, Fig. 4A). Time and the interaction of time x treatment were not significant (F ≤ 0.48, *P* ≥ 0.49).

Bees in the light treatment experienced more disruptive contacts than bees in the dark treatment (*F*_1,44_ =9.44, *P* = 0.004, Fig. 4B). Time had no significant effect (*F*_1,44_ =1.85, *P* = 0.18), but there was a significant interaction of time x treatment (*F*_1,34_ =48.53, *P* < 0.001) because bees in the light treatment experienced more disruptive contacts. On average, within a 5 min interval, a sleeping bee received 1.09 ± 2.24 contacts from non-sleeping nestmates in the light treatment and 0.31 ± 0.97 contacts from non-sleeping nestmates in the dark treatment (3.5-fold more disruptive contacts in the light as compared to the dark treatment).

## Discussion

In the typical dark inside colonies, honey bees demonstrate a stable circadian sleeping rhythm. However, constant light decreased honey bee sleep after roughly 79 h and led to behavioral alterations such as increased sleeping in dimmer areas and frequent physical disturbances from non-sleeping bees. In addition, exposure to constant light was associated with greater distortion of the free running period (τ) from the 12 h light: 12 h dark and constant groups, showing consistent trends by several measures. These also align with previous findings of longer period and increased circadian rhythm instability under constant light. Our results match those of Moore and Rankin^30^ who reported that European honey bee foragers had a longer circadian free-running period of locomotor activity under constant light as compared to constant dark conditions. The authors also found that bees under constant light had higher probabilities of displaying arrhythmic activity than individuals under constant dark. Moore and Rankin^31^ showed that bees can entrain their locomotor rhythms to several light: dark and temperature cycles. Fuchikawa and Shimizu^32^ also confirmed this result in Japanese honey bees (*Apis cerana japonica*). Our results thus suggest that constant light exposure could be detrimental to bees, leading to sleep loss and reduced sleep quality via disruption from nestmates.

In our experiment, bees were subjected to multiple days of dark or light. In nature, foragers can remain inside their nests for extended periods because of poor weather, particularly rainfall or cold, with the most extreme examples occurring in overwintering colonies^25^. Thus, it is reasonable to expect that foragers have evolved to cope with extended periods of darkness, and we confirmed that, at least on a scale of five days, forager sleep was not disrupted.

We did not periodically remove dead bees or change out their sucrose solution, as is standard practice with caged bee studies^33^, to avoid providing a zeitgeber that could alter circadian rhythms. The presence of dead bees may have impacted the sleep behavior of living nestmates. The trials were thus limited to five days. A longer trial period—provided that the experimenters can provide fresh food and remove dead bees without introducing potential time information to synchronize or alter bee circadian rhythms—could provide greater insight into how constant light disrupts bee circadian rhythms. Another limitation of our study is that we ran eight trials with 16 different colonies and 281 bees. A larger number of trials could have allowed us to run more detailed analyses on the effects of constant light on bee sleep rhythms. However, we were still able to identify a clear disruptive effect of light on sleep (*P* = 0.0017, Fig. 2), trends in changed sleep rhythmicity (Fig. 3), and the behavioral disruption of sleep (jostling, *P* = 0.004, Fig. 4B). As predicted, the bees also preferred to sleep in the darker part of their cages (Fig. 4A). In the dark condition, these contacts were higher before 96 h and then decreased to 0, when the disruptive contacts significantly increased for the constant light bees. This likely occurred because dark condition bees, at this time point, were in a non-sleep phase (Fig. 2B), and thus there were very few sleeping bees to be disrupted by other bees. Although light treatment bees were sleeping during this time period, the movements of other bees that disrupted their sleep appear only to have increased after 90 h. We speculate that the loss of normal circadian sleep rhythms contributed to the behavior of these “sleep disruptor” bees.

Given our findings, future studies may focus on the effects of artificial light on the clock genes expressed in bee brains^34^ and how exposure to constant light may alter foraging patterns^18^. If foraging efficiency declines, colony fitness should also decrease. In addition, the stressful effects of sleep deprivation should be explored. Klein et al.^35^ found that sleep restriction in honey bees significantly lowers their waggle dance directional precision and thereby lowers the foraging efficiency of signal receivers. Fruit flies like *Drosophila melanogaster* under constant artificial light have been shown to have a significant decrease in fecundity rates and adult survival^36^. Frequent or repeated exposures to light at night, which could occur with growing light pollution in urban areas and could be exacerbated by bee bearding, the clustering of bees outside the nest on days and nights with abnormally high temperatures, may be chronic stressors that, like other chronic stressors, can lead to changes in animal performance (e.g. growth, endurance, disease resistance) and behavioral patterns (e.g. feeding, aggression, reproduction)^37^. Studies that have measured different honey bee behavioral and physiological stress responses, including cellular stress responses^38^ may be a useful model for studying different ways in which constant light exposure affects honey bee behavior and physiology. Sauer et al.^39^ have shown that honey bees can compensate for a sleep deficit by intensifying their sleep. However, the degree to which such sleep compensation can correct for extended sleeplessness induced by constant light remains to be determined.

Studying the effects of ALAN on honey bee swarms in the field, or non-enclosed colonies would be valuable, particularly in urban settings. In addition, there is an increase in urban beekeeping^40^. This surge in urban colonies could potentially expose a larger proportion of bee populations to constant artificial light. The urbanization and industrialization of human populations have led to a rise in ailments related to circadian disruption. This urbanization of pollinators presents a significant, potentially worrying, and yet largely unexplored environmental challenge to honey bees. It may also be useful to consider how other honey bee species, like *Apis dorsata* and *Apis florea*, which typically nest in the open, can cope with artificial light at night. Although artificial light has not played a role in the evolutionary history of these species, they are subject to periodic bright moonlight, depending upon their hive locations. Understanding their behavioral and physiological adaptations to light at night could inform our broader understanding of the impact of ALAN on bee populations.

Finally, we should consider how to mitigate the adverse effects of artificial light at night by designing more wildlife-friendly lighting solutions that reduce light pollution. There have been recent strides to develop lighting that minimizes harm to insects and can provide solutions for new urbanized areas^41^. Incorporating such ecological considerations into our urban planning strategies could potentially safeguard the well-being of multiple pollinators and ensure the sustainability of urban ecosystems.

## Data Availability

Data will be available upon publication at Zenodo.com at the DOI: 10.5281/zenodo.12908855.
